# Can men be trusted in population-based surveys to report couples’ medical care for infertility?

**DOI:** 10.1186/s12874-018-0566-y

**Published:** 2018-10-19

**Authors:** Soraya Belgherbi, Elise de La Rochebrochard

**Affiliations:** 1 Université Paris-Saclay, Univ. Paris-Sud, UVSQ, CESP, INSERM, Kremlin-Bicêtre, France; 2 Institut national d’études démographiques (INED), F-75020 Paris, France

**Keywords:** Survey methodology, Respondents, France, Male, Female, Infertility, Assisted reproductive technologies

## Abstract

**Background:**

Men are usually excluded from surveys on reproductive health as some works have cast doubts on their ability to accurately report information on reproduction. Recent papers challenged this viewpoint, arguing that the quality of men’s reports depends strongly on use of an appropriate study design. We aimed to explore the relevance of evaluating couples’ use of medical care for infertility based on men’s interviews in a population-based survey.

**Methods:**

The study was based on the last French sexual and reproductive health study (Fecond) conducted by phone interviews among a population-based sample of 2863 men and 4629 women aged 20–49 years.

**Results:**

Among respondents who had ever tried to have a child, the use of infertility medical care by couples (i.e. by the respondents and/or their partners) within the previous 15 years was 16% (95%CI 14 to 18%) based on men’s reports and 17% (95%CI 15 to 18%) based on women’s reports (*p* = 0.43). Men’s and women’s reports were remarkably concordant on most items (infertility duration, treatment). The main discrepancy concerned male medical checkup, which was reported much more often by male respondents than female respondents (86% vs. 57%, *p* < 0.001 for sperm analysis, 56% vs. 27%, *p* < 0.001 for male genital examination).

**Conclusions:**

It is time to trust men to report couples’ infertility medical care in reproductive surveys, as they provide information remarkably concordant with that provided by women. Conversely, women may poorly report the infertility checkups of their male partner.

## Background

Infertility is still often perceived as “*women’s business*” [1, 2]. Social representations have constantly designated women as being accountable for childlessness [3, 4]. After 1910, it was medically recognized that sexually potent men can contribute to infertility [5]. Several works have since revealed a marked variability of sperm count that can be affected by several factors [6]. A passionate debate has arisen regarding a severe decrease of sperm count and more generally regarding an impairment of male reproductive health that has been called the “testicular dysgenesis syndrome” [7, 8]. Other works have claimed that the reproductive biological clock affects not only women through the menopause but also men through a phenomenon controversially called the “andropause” [9–11]. Meanwhile, medical recognition of the male contribution to couples’ infertility has constantly increased whereas the contribution of unexplained infertility decreased [12]. Male factors are now recognized as contributing to more than half of all cases of infertility [2, 13, 14]. Despite these advances, it is still a common social belief that women are the (only) one to blame when a couple has difficulties in having a child [3, 15, 16]. Much remains to be done to overcome outdated traditional gender stereotypes [1, 4, 12, 17].

Whatever the origin of infertility, it is the woman’s body that is treated through assisted reproductive technologies (ARTs). Even to overcome male infertility, medical help targets the female partner to obtain a pregnancy [12]. The role of the male partner in reproductive care is so limited that he has been described in the literature as an “*onlooker*”, a “*bystander*”, the “*spare part*” or the “*second sex in reproduction*” [1, 16–20]. In line with this medical logic, most research has considered that infertility medical care is a “*woman’s medical story to tell*” [1, 12]. Even in psychosocial research on infertility, men are so marginally considered that the question has been raised: “*Where are all the men?*” [1].

Men are excluded not only from the medical pathway but also from surveys on reproductive health. Firstly, in line with gender stereotypes, researchers often believe that men would be reluctant to participate in such studies [21]. Secondly, some works have cast doubts on the ability of men to accurately report information on reproduction, even for very basic events such as pregnancies, livebirths and their timing [22, 23]. Comparing answers given by each partner, an American study concluded that men cannot be trusted to report reproductive histories [22]. However, recent papers challenged this viewpoint, arguing that the quality of men’s reports depends strongly on use of an appropriate study design [1, 24]. For men to be effectively involved in reproductive surveys, it has been suggested that men only should be recruited, without interviewing their female partners, in order to overcome the traditional secondary role of males [1, 25]. This methodology was successfully implemented in the American National Survey of Family Growth with a participation rate of 75% among men versus 78% among women [26].

We aimed to investigate whether men and women provide concordant information on infertility medical care when interviewed in a large population-based survey recruiting men independently (the male respondents were not the partners of the female respondents).

## Methods

The Fecond (Fertility, Contraception and Sexual Dysfunction) study is a population-based survey conducted in France in 2010. It received institutional review board approval from the French Data Protection Authority (authorization CNIL no. 2009–674). The study methodology has been described in detail elsewhere [27]. This study aimed to explore several sexual and reproductive health issues including infertility medical care.

### Sampling methodology and selection of the study population

**Fig. 1:**
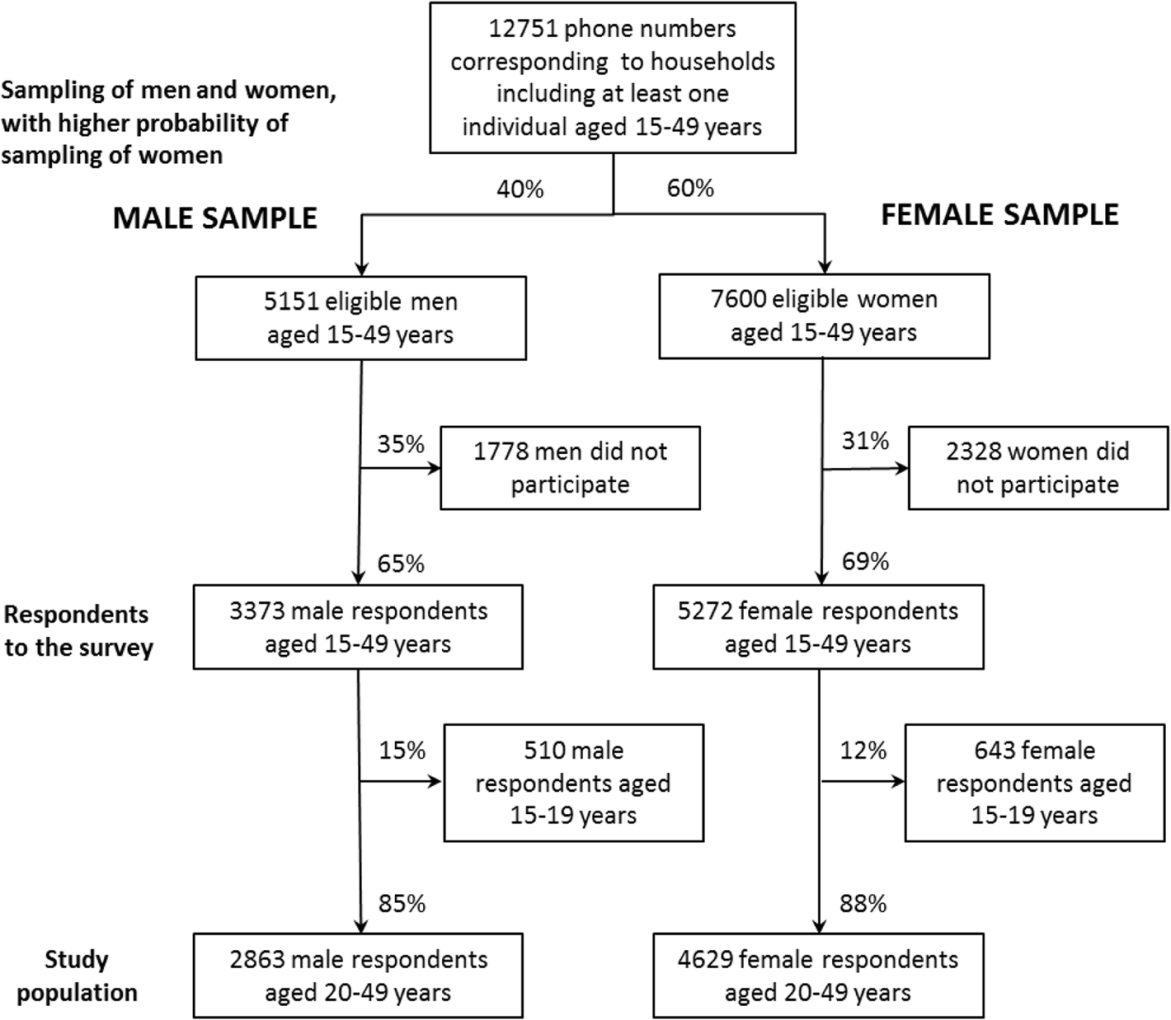
Flow chart of sampling methodology and selection of the study population

Recruitment in the survey was based on two-stage stratified sampling (Fig. 1). The first sampling stage generated 12,751 representative households by random digit dialing methodology of landline and mobile phone numbers. The second sampling stage selected one person aged 15–49 per household using the Kish method. Considering current doubts as to the feasibility of collecting good-quality data on sexual and reproductive health from men, it was decided to use a higher probability of inclusion for women (60%) than for men (40%) to ensure a larger female sample. To increase participation, a call-back strategy was developed and has been detailed elsewhere [27]. The participation rate was 65% among men and 69% among women. A total of 3373 men and 5272 women participated in the survey by responding to an anonymous telephone interview lasting on average 41 min. To explore use of medical care for infertility, the study population was restricted to 2863 men and 4629 women of reproductive age (20–49 years).

### Data collection

The questionnaire collected information on sociodemographic characteristics and on sexual and reproductive health. Detailed information was collected on attempts to have a child (ever if the attempt was successful with the birth of a child or if it was not (yet) successful), on pregnancies (ever planned or unplanned) and their outcomes. Based on this comprehensive information, respondents were classified in two groups: those who had tried at least once in their life to have a child (“ever tried to have a child”) and those who had never tried to have a child. Respondents who had never tried to have a child were not potentially exposed to infertility.

A section of the questionnaire was dedicated to infertility medical care received by the respondents and/or their partners. When the respondents declared that they and/or their partners had had infertility medical care, a detailed questionnaire investigated the couple’s medical pathway more closely. In order to limit memory bias, this detailed questionnaire was only completed when the first consultation for infertility dated from 15 years previously or less.

Identical questions were put to both male and female respondents, the wording being adapted to the respondent’s gender.

### Statistical analyses

All analyses were performed using Stata 13.0 software (StataCorp). The concordance between male respondents’ reports and female respondents’ reports was tested. As male and female respondents were independently recruited, men and women were compared using statistical tests for independent observations.

All percentages were weighted to account for the complex sampling design. Firstly, respondents were assigned a sampling weight, inversely proportional to the probability of being selected in the sample. Then, using census data, post-stratification adjustments were applied to reflect the sociodemographic structure of the target population. Finally, the weights were adjusted to the actual sample size. Following guidelines for analysis of complex sample survey design [28, 29], weighted percentages were compared using the χ^2^ test with the subpopulation option to correctly estimate the standard errors.

## Results

Based on male respondents’ reports, in the general population, 11% of couples used infertility medical care versus 16% based on female respondents’ reports (*p* < 0.001) (Table 1). However, only 53% of male respondents and 66% of female respondents (*p* < 0.001) had ever tried to have a child, so a large proportion of respondents (47% for men and 34% for women) were in fact outside the scope of infertility. Considering only respondents who ever tried to have a child (Table 2), 16–17% of couples used infertility medical care within the previous 15 years based on male and female reports (*p* = 0.43).

**Table 1:** Reproductive status and use of infertility medical care among respondents aged 20–49 years old^a^

	Respondent’s gender		*P*-Value ^b^
	Male	Female	
Ever tried to have a child	53% (51–55)	66% (65–68)	< 0.001
Ever used medical care for infertility^c^	11% (10–12)	16% (15–17)	< 0.001
Used medical care for infertility within the previous 15 years^c^	8% (7–10)	11% (10–12)	< 0.001
Ever treated for infertility^c^	6% (5–7)	9% (8–10)	< 0.001

^a^All percentages are weighted with 95% confidence intervals in brackets

^b^*P*-value of the chi-square test comparing the female and male respondents’ distributions. To take into account the complex sampling design of the Fecond survey, the subpopulation option in Stata® was used for correct calculation of the standard errors of the estimates

^c^Either the respondents and/or their partners

**Table 2:** Use of infertility medical care among respondents who ever tried to have a child^a^

	Respondent’s gender		*P*-Value ^b^
	Male	Female	
Ever used medical care for infertility^c^	21% (18–23)	24% (22–26)	0.04
Used medical care for infertility within the previous 15 years^c^	16% (14–18)	17% (15–18)	0.43
Ever treated for infertility^c^	12% (10–14)	13% (12–15)	0.16

^a^All percentages are weighted with 95% confidence intervals in brackets

^b^*P*-value of the chi-square test comparing the female and male respondents’ distributions. To take into account the complex sampling design of the Fecond survey, the subpopulation option in Stata® was used for correct calculation of the standard errors of the estimates

^c^Either the respondents and/or their partners

Among couples who had infertility medical care within the previous 15 years (Table 3), both partners had consulted in about two of three cases, based on male and female reports (70% vs. 63%, *p* = 0.12). Male and female reports both indicated that medical help was sought at less than 12 months of infertility in about 2 of 5 cases (42% vs. 39%, *p* = 0.32). Female respondents usually had their first consultation for infertility with a gynecologist (84%). Male respondents also mainly first saw a gynecologist (65%).

**Table 3:** Medical care pathway for infertility^a^

	Respondent’s gender		*P*-Value^b^
	Male	Female	
Who used medical care for infertility in the couple			0.12
Woman alone	26% (19–34)	35% (30–40)	
Man alone	4% (2–9)	2% (1–6)	
Woman and man including:	70% (62–77)	63% (58–68)	
Together at the first consultation	52% (45–60)	38% (33–43)	
Woman first and the man later	17% (12–22)	24% (20–28)	
Man first and the woman later	0% (0–3)	1% (0–3)	
Don’t know in which order	0% (0–3)	1% (0–2)	
Age of the respondent at the first consultation^c^			0.16
< 25 years	8% (4–13)	16% (12–20)	
25–29 years	41% (33–49)	38% (33–43)	
30–34 years	33% (27–41)	30% (25–35)	
35–39 years	13% (9–19)	12% (9–17)	
≥ 40 years	5% (3–9)	4% (3–8)	
Length of couple’s infertility at the first consultation			0.32
≤ 11 months	42% (35–50)	39% (35–45)	
12 months	21% (16–28)	28% (24–33)	
13 to 24 months	26% (19–34)	21% (17–26)	
≥ 25 months	10% (6–16)	11% (8–15)	
First doctor consulted by the respondent^d^			< 0.001
General practitioner	21% (15–29)	12% (9–16)	
Gynecologist	65% (56–72)	84% (80–88)	
Other specialty	14% (9–21)	4% (2–6)	
Couple’s infertility checkup^d^			0.18
None	2% (0–7)	4% (3–7)	
At least one exam including:	98% (93–100)	96% (93–97)	
Temperature curve	66% (57–74)	64% (58–69)	0.65
Gynecologic examination	94% (87–97)	92% (89–95)	0.62
Invasive female examination^e^	42% (33–51)	45% (40–51)	0.52
Post-coital test or cervical mucus	32% (24–41)	34% (29–39)	0.78
Male genital examination	56% (48–65)	27% (22–32)	< 0.001
Sperm analysis	86% (80–91)	57% (52–62)	< 0.001
Hormonal checkup	81% (73–88)	64% (59–69)	< 0.001
Sexual transmitted infection testing	61% (52–70)	52% (46–57)	0.08
Origin of the couple’s infertility^d^			0.12
Female	22% (16–30)	29% (25–34)	
Male	12% (7–18)	7% (5–10)	
Mixed (male and female)	5% (2–10)	3% (2–5)	
Unspecified	61% (53–69)	61% (56–66)	
First doctor supportive with respondent^d^			0.83
Absolutely	57% (48–66)	59% (53–64)	
Rather	26% (19–35)	24% (19–29)	
No	16% (11–24)	18% (14–22)	
Topic(s) covered with the respondent during medical care^d^			
Sexual life	47% (38–55)	54% (48–59)	0.17
Different treatments of infertility	64% (55–71)	58% (53–63)	0.29
Adverse effects of treatments	46% (38–55)	43% (38–48)	0.55
Adoption	18% (13–26)	15% (12–20)	0.44
None of these topics	25% (18–33)	21% (17–25)	0.35

^a^Among respondents who used medical care for infertility themselves and/or their partners with a first consultation dating from 15 years previously or less. All percentages are weighted with 95% confidence intervals in brackets

^b^*P*-value of the chi-square test comparing the female and male respondents’ distributions. To take into account the complex sampling design of the Fecond survey, the subpopulation option in Stata® was used for correct calculation of the standard errors of the estimates

^c^Age of the respondent at the time of the couple’s first consultation, whoever (first) consulted, the respondent or his/her partner

^d^Item asked only if the respondent had consulted him/herself

^e^Invasive female examination included hysterosalpingogram, hysteroscopy and laparoscopy

Male and female respondents who used infertility medical care agreed that in nearly every case at least one checkup examination was performed (96 vs. 98%, *p* = 0.18). Male and female reports agreed concerning the frequency of each checkup examination, except for three investigations (men’s genital examination, sperm analysis, hormonal test), more frequently cited by male respondents. The origin of infertility was unspecified for most male and female respondents (61%). Men and women usually found the first doctor “absolutely” supportive (57% vs. 59%, *p* = 0.83). Moreover, male and female respondents agreed on topics covered during medical care.

Among couples treated for infertility (i.e. the respondents and/or their partners had been treated) within the previous 15 years, male and female respondents agreed on the frequency of use of each specific treatment and on treatment outcome (Table 4). Nearly all couples had ovarian stimulation (84% vs. 87%, *p* = 0.48). For the majority (70% vs. 73%, *p* = 0.77), this was the first treatment. In vitro fertilization (IVF) was the second most frequent treatment received by around one-third of the treated population (36% vs. 31%, *p* = 0.31). Nearly three of five treated couples achieved a birth following treatment (58% vs. 59%, *p* = 0.75). Fewer men than women declared that treatment was a “very difficult” experience (14% vs. 29%, *p* = 0.01).

**Table 4:** Use and outcome of medical treatments for infertility^a^

	Respondent’s gender		*P*-value^b^
	Male	Female	
Treatment(s) used by the couple			
Ovarian stimulation	84% (76–90)	87% (82–91)	0.48
IVF^c^	36% (28–45)	31% (25–37)	0.31
Artificial insemination with husband sperm	28% (21–37)	24% (19–30)	0.41
Artificial insemination with donor sperm	5% (2–11)	3% (1–6)	0.22
Surgical intervention	13% (7–24)	13% (9–19)	0.98
Other treatment	23% (16–32)	22% (17–28)	0.78
First treatment used by the couple			0.77
Ovarian stimulation	70% (59–79)	73% (67–79)	
IVF^c^	5% (2–10)	3% (2–6)	
Artificial insemination with husband sperm	3% (1–7)	3% (1–8)	
Artificial insemination with donor sperm	1% (0–7)	0% (*n* = 0)	
Other	21% (13–32)	20% (15–26)	
Last treatment used by the couple^d^			0.68
Ovarian stimulation	36% (27–45)	44% (37–51)	
IVF^c^	24% (17–33)	21% (16–27)	
Artificial insemination with husband sperm	12% (7–19)	13% (9–18)	
Artificial insemination with donor sperm	2% (1–9)	2% (1–5)	
Other	26% (17–37)	20% (15–27)	
Outcome of couple’s treatment(s)			0.75
Birth	58% (48–67)	59% (52–66)	
No birth including:	42% (33–52)	41% (34–48)	
Treatment(s) stopped	29% (21–38)	29% (23–36)	
Treatment(s) ongoing at time of survey	14% (8–22)	12% (8–17)	
Miscarriage during treatment(s)	24% (17–33)	19% (14–25)	0.32
Treatment experiences of the respondent			0.01
Very difficult	14% (9–21)	29% (23–35)	
Difficult	37% (28–47)	38% (31–44)	
Not difficult	49% (39–59)	34% (27–41)	
Treatment consequences in the respondent’s life			
Disturbances in sexual life	38% (29–48)	44% (37–51)	0.33
Disturbances in couple’s life	38% (30–48)	45% (38–51)	0.29
Disturbances in social life	19% (13–27)	29% (23–35)	0.05
Disturbances in professional life	22% (14–33)	24% (19–30)	0.76
No disturbance	42% (33–52)	39% (33–46)	0.65

^a^Among respondents who had been treated for infertility themselves and/or their partners following a first consultation for infertility dating from 15 years previously or less. All percentages are weighted with 95% confidence intervals in brackets

^b^*P*-value of the chi-square test comparing the female and male respondents’ distributions. To take into account the complex sampling design of the Fecond survey, the subpopulation option in Stata® was used for correct calculation of the standard errors of the estimates

^c^IVF: in vitro fertilization including IVF with intracytoplasmic sperm injection (ICSI)

^d^If only one treatment was used, then the last treatment is also the first treatment

## Discussion

In a large representative sample of women and men, we explored use of infertility medical care by couples (the respondents and/or their partners). Based on female respondents, 16% of couples used medical care for infertility in the French population, a frequency consistent with estimations in other developed countries [2, 26, 30]. On the other hand, based on male respondents, only 11% of couples used medical care for infertility, which is about one-third lower than the estimation based on female respondents (16%, *p* < 0.001). A very similar gender gap was observed in the 2006–2010 National Survey of Family Growth in the USA, with use of infertility services estimated at 17% based on female reports versus 9% based on male reports [26]. When restricting use of medical care for infertility to treatment within the previous 15 years, it remained about one-third lower when declared by men (8%) than when declared by women (11%). There was thus no evidence that the gender gap could be explained by differences in gender-related long-term memory bias. At first sight, the gender discrepancy seemed to be in agreement with results claiming that men cannot be trusted on reproductive topics [22]. However, deeper investigation showed that this apparent discrepancy resulted largely from confusion bias.

Using classic epidemiological methodology [31], the use of infertility medical care was estimated in the exposed population. In this study, to be exposed to infertility, a person must have tried to have a child. Exposure to infertility was thus defined based on the respondent’s fertility intentions and not on his/her reproductive behavior (having unprotected intercourse). An American study demonstrated the gap between the two approaches, as some women had unprotected intercourse but did not try to have a child; they were “okay either way” about getting pregnant and in consequence had a very low level of infertility medical care [32, 33]. When considering respondents having “ever tried to have a child”, male respondents of our French study were much less exposed to the risk of infertility than female respondents (47% vs. 34%, *p* < 0.001). When considering male and female respondents who had ever tried to have a child (the group exposed to infertility), infertility medical care was much more frequent (21–24%) than in the general population (11–16%). Moreover, male and female reports were quite concordant (21% vs. 24%, *p* = 0.04). The concordance between male and female reports was even more striking when examining only use of medical care within the previous 15 years (16% vs. 17%, *p* = 0.43). To conclude, the lower level of infertility care declared by men was primarily mediated by higher male probability of never having been exposed to infertility (never having tried to have a child).

To discuss gender differences in infertility care, we need to understand why fewer men ever tried to have a child. A first explanation could be that men tend to declare less often than women that they tried to have a child, i.e. a larger gap between intentions and behavior among men, possibly because they may more often be “okay either way” about pregnancy. However, the gap was probably largely explained by gender differences in demographic and sociological phenomena. In France, 14% of women, but 21% of men, finally remain childless [34]. Nearly all women and men (95%) would like to become a parent [2, 35], but not everyone has the opportunity to try to have a child. The major barrier to parenthood is to have never lived in a couple relationship, with strong social inequalities in the “game of love” for men [36]. Socially disadvantaged men have difficulties in finding a partner, whereas disadvantaged women do not face such “exclusion from the marriage market” [36, 37]. Conversely, socially advantaged men may have children with different female partners, with a growing tendency to divorce and re-partnership [38]. These gender differences in partnership pathways have a direct impact on the lower proportion of men who have ever tried to have a child and thus have ever been at risk of infertility.

Focusing more specifically on respondents who had used infertility medical care within the previous 15 years, we demonstrated a high level of concordance between male and female respondents. Male respondents declared that the couple’s first consultation for infertility (the first consultation of whoever consulted first, the woman and/or the man) occurred at an early stage (less than 1 year of infertility) in 42% of cases and at a late stage (more than 2 years of infertility) in 10% of cases, which is in line with female respondents’ declarations (39% and 11%, respectively). The high frequency (39–42%) of early use of infertility medical care is in line with the hypothesis that couples have moved from “*silence to impatience*” on the infertility issue [39]. The “give time to time” approach could be less acceptable for couples in a society where ARTs are increasingly used [40, 41].

Regarding information on who had sought medical help for infertility, male and female reports were not significantly different (*p* = 0.12), although male respondents tended to declare a higher frequency of male partner medical care. Male and female reports were less concordant on who first sought medical help: male respondents were more likely than female respondents to declare that both partners were present at the first consultation (52% vs. 38%). This probably reflects difficulties in distinguishing between the first consultation for infertility and a routine female gynecological checkup during which the issue of infertility had been discussed. The first doctor consulted by the female respondents was usually a gynecologist (84%). For their first consultation, male respondents also very often consulted a gynecologist (65%). It is likely that this gynecologist was their female partner’s gynecologist and that the female partner was also present at this consultation. Further research would be needed to examine whether a consultation with the female partner’s gynecologist is the most efficient way to include men in infertility care. The need to improve accessibility and medical care for men in sexual and reproductive health services has already been emphasised [42].

With regard to infertility checkup, male and female respondents agreed that at least one investigation had been performed in nearly all couples who sought medical help for infertility (96–98%). Male reports on female partner checkup were remarkably concordant with female reports: 94% (vs. 92%, *p* = 0.62) reported a gynecologic examination, 66% (vs. 64%, *p* = 0.65) a temperature curve, 42% (vs. 45%, *p* = 0.52) an invasive female examination (hysterosalpingogram, hysteroscopy or laparoscopy) and 32% (vs. 34%, *p* = 0.78) a post-coital test or cervical mucus examination.

With regard to the two investigations specifically concerning the male partner, there was major discrepancy between male and female reports: 86% of male respondents reported sperm analysis versus only 57% of female respondents (*p* < 0.001) and 56% of male respondents reported male genital examination versus only 27% of female respondents (*p* < 0.001). Other investigations that could concern both partners also tended to be more frequently cited by male respondents than by female respondents: hormonal checkup (81% vs. 64%, *p* < 0.001) and sexual transmitted infection testing (61% vs. 52%, *p* = 0.08). To advance our knowledge, it would have been interesting to cross results on medical checkup with the origin of infertility. Unfortunately, the origin of infertility was very poorly reported by both male and female respondents, as 61% did not declare any specific origin of their infertility. Our results are in line with those of the American National Survey of Family Growth which showed that male partners reported a higher proportion of male examination (82%) than female partners (72%) [43]. This raises the question of whether women can be trusted to declare male infertility examination. The gender gap is so large that we may wonder whether female respondents are not always aware that their male partner has undergone genital examination or sperm analysis. In a Canadian qualitative study, diagnosis of male infertility was often followed by a period of male denial sometimes lasting several years, while the man let his female partner pursue medical testing for female factors of infertility [44]. Denial is probably linked to strong stigmatization of male infertility, often interpreted as proof of male impotence [4, 5, 15, 16, 45]. Because infertile men are ridiculed in society [15, 16, 18], some women prefer to unfairly take the “blame” for the couple’s infertility to protect their male partner from thoughtless or hurtful comments and jokes from family, friends and colleagues [2, 4, 16, 18]. In this context, diagnosis of male infertility can be perceived as threatening the man’s masculinity and infertile men exhibit strong feelings of failure, shame and guilt [16, 18, 46]. Male loss of self-esteem is reflected in the terms used by infertile men to describe themselves, such as “*emasculated*”, “*eunuch*”, “*loser*” or “*garbage*” [44, 45]. Further research would be needed to confirm the hypothesis that men more reliably report male infertility checkup than their female partner, for example by conducting qualitative studies with semi-directive interviews including both partners of the same couples to explore the interaction and logics of the two partners.

Among couples treated for infertility, male and female reports were remarkably concordant, giving very similar pictures of treatment histories. Ovarian stimulation was the first-line treatment, undergone by 84–87% of couples. IVF had been used by 31–36% and husband artificial insemination by 24–28%. At the survey time, 58–59% of treated couples had succeeded in having a child and 12–14% were still being treated and may have had a child later. Considering only respondents who had undergone IVF (those with the most severe infertility), the long-term success rate was 47–52% (men’s and women’s reports, *p* = 0.64). This rate is identical to that (48%) observed in another study on long-term outcome of patients treated in eight French IVF centers [47].

In our study, among respondents treated for infertility, 66% of female respondents and 51% of male respondents declared that treatment experiences were (very) difficult (*p* < 0.01), in line with other research showing greater reported distress in women than men [48–50]. The greater distress among women is linked to the fact that they are first in line, as infertility treatments affect the female body. However, this gender difference could also reflect a tendency to comply with traditional gender stereotypes, women being expected to voice their sadness whereas men are supposed to play the “*emotional rock*”, the “*committed and supportive partner*” [4, 17, 19, 51, 52].

## Conclusions

To conclude, it is time to trust men to report infertility medical care in reproductive surveys. The information they provided is remarkably concordant with that based on women’s reports. Male reports were of high quality even when relating to female partner checkup. Conversely, female respondents may poorly report the infertility checkups of their male partner. Further research is needed on this issue. We demonstrated that gender differences in use of infertility medical care were strongly mediated by differences in opportunities to try to have a child. As men and women have different reproductive pathways, it is important to include men in reproductive health research.

## Abbreviations

ARTs: Assisted Reproductive Technologies
CI: Confidence Interval
CNIL: Commission Nationale de l’Informatique et des Libertés (French data protection authority)
Fecond: Fécondité-Contraception-Dysfonctions sexuelles (French survey. Translation: Fertility-Contraception-Sexual Dysfunction)
IVF: In Vitro Fertilization
USA: United States of America
